# Functional Adrenal Collision Tumor in a Patient with Cushing's Syndrome

**DOI:** 10.1155/2020/7415762

**Published:** 2020-12-14

**Authors:** Cathy Zhou, Ghaneh Fananapazir, Michael J. Campbell

**Affiliations:** ^1^Department of Radiology, University of California, Davis Medical Center, Sacramento, CA, USA; ^2^Department of Surgery, University of California, Davis Medical Center, Sacramento, CA, USA

## Abstract

Adrenal collision tumors are rare and produce unique diagnostic challenges for clinicians. We report the case of a 45-year-old woman with obesity and diabetes mellitus and an incidentally-discovered adrenal mass containing macroscopic fat, thought to be a myelolipoma. A functional workup confirmed adrenocorticotropic hormone- (ACTH-) independent Cushing's syndrome. The patient underwent a successful laparoscopic adrenalectomy with pathology showing an adrenal collision tumor consisting of an adrenocortical adenoma and a myelolipoma. Postoperatively, the clinical symptoms, body mass index, and hemoglobin A1C all improved. Clinicians should consider a functional workup in patients with radiographically diagnosed myelolipomas as some may prove to be hormonally active collision tumors.

## 1. Introduction

“Adrenal collision tumor” is the radiographic term used to describe the appearance of a single radiographic mass consisting of two histologically distinct adrenal neoplasms. Adrenal collision tumors involving a myelolipoma are particularly challenging because radiologists are trained to label adrenal lesions with any amount of macroscopic fat as myelolipomas [1]. Clinicians are trained that radiographically diagnosed myelolipomas do not need further imaging or a biochemical evaluation for autonomous hormone production like other adrenal tumors. We report a case of a 45-year-old woman with Cushing's syndrome secondary to an adrenal tumor diagnosed as a myelolipoma on abdominal imaging. Ultimately, the patient was found to have an adrenal collision tumor composed of a functioning adrenocortical adenoma and a myelolipoma. Only five similar cases have been previously described and are reviewed in this report [2–6].

### 1.1. Case Presentation

**Figure 1:**
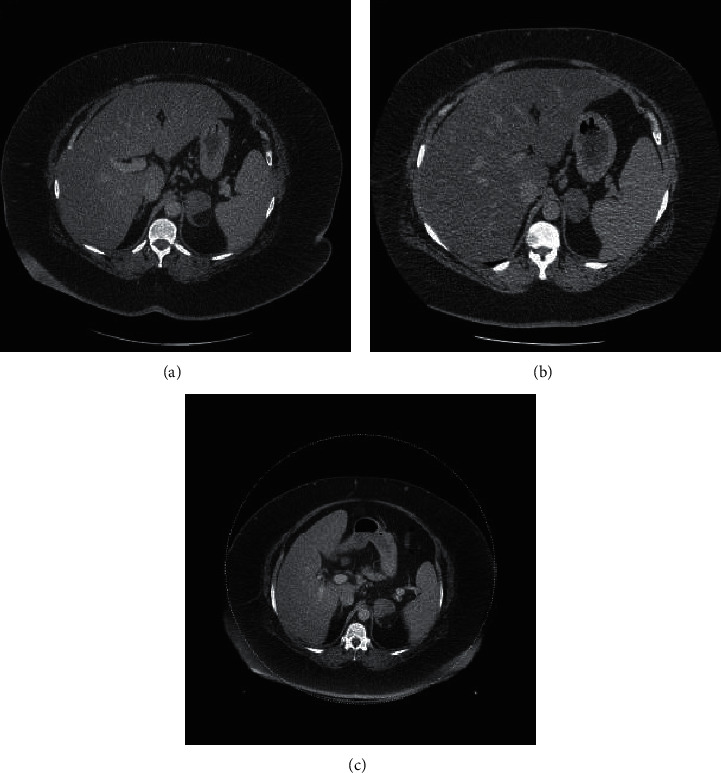
A CT for abdominal pain in November 2012 revealed a presumed adrenal myelolipoma with a focus of macroscopic fat (a). In comparing to a prior exam from April 2007 (b), this mass had grown in size, and subsequent imaging from April 2016 (c) continued to demonstrate growth.

In November 2012, a 45-year-old Caucasian woman presented to her primary care physician with lower abdominal pain. At the time of presentation, her medical history was notable for obesity (weight = 183.2 kg and body mass index (BMI) 61.6 mg/m^2^), type 2 diabetes mellitus (hemoglobin A1C (HbA1C) = 7.5%), hypertension, and recurrent episodes of cellulitis. A contrast-enhanced CT scan of the abdomen and pelvis in the portal venous phase was obtained that showed no source for her abdominal pain but did note an incidental 5.5 × 4.0 cm left adrenal tumor containing macroscopic fat and reported as a myelolipoma (Figure 1(a)). This tumor had grown from a CT scan in April 2007, where it measured 4.4 × 3.3 cm (Figure 1(b)). The right adrenal appeared normal on both scans.

In September 2014, the patient was referred to an endocrinologist for worsening diabetes mellitus. At this visit, her weight was 184.7 kg with a BMI of 61.9 mg/m^2^ and HbA1C of 9.2%. Because of clinical concern for hypercortisolism, a functional workup was initiated. A 1 mg dexamethasone suppression test revealed a postsuppression cortisol of 9.3 *µ*g/d (reference range <1.8 *µ*g/dL). Serum aldosterone was 4.7 ng/dL with a renin activity of 0.3 ng/ml/hr. Plasma free metanephrines were less than the upper limit of normal, and a morning adrenocorticotropic hormone (ACTH) was 16 ng/L (reference range 6–50 ng/L). The patient was lost to follow-up before completing further testing.

In June 2015, she reestablished care with her endocrinologist. Physical exam was notable for obesity, but without other clinical signs of Cushing's syndrome, including moon facies, buffalo hump, or striae. A dual-energy X-ray absorptiometry (DEXA) scan showed normal bone health. The 24-hour urine cortisol level was 54.1 *µ*g/day (reference range <45 *µ*g/day). Repeat 24-hour urine cortisol was again elevated at 47.5 *µ*g/day. ACTH was 8 ng/L (reference range 6–50 ng/L) and dehydroepiandrosterone sulfate (DHEAS) was suppressed at <15 *µ*g/dL (reference range 32–240 *µ*g/dL). Repeat 1-mg dexamethasone suppression test was elevated at 13.5 *µ*g/dL (reference range <1.8 *µ*g/dL). BMI was 62.4 kg/m^2^ (weight 186.1 kg), and HgA1C was 9.0%. Given continued symptomatology and positive lab values, the patient was then referred to endocrine surgery with suspicion for Cushing's syndrome from her left adrenal myelolipoma but failed to make her appointments.

**Figure 2:**
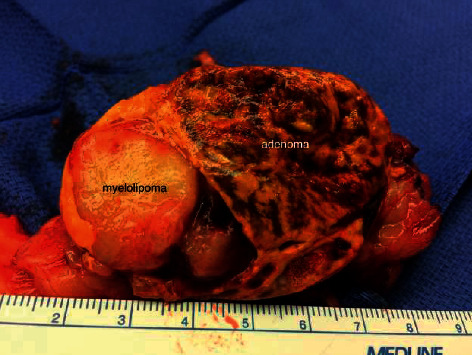
The surgical specimen demonstrates the characteristic macroscopic fat component of the myelolipoma with an adjacent well-circumscribed solid golden yellow adenoma.

**Figure 3:**
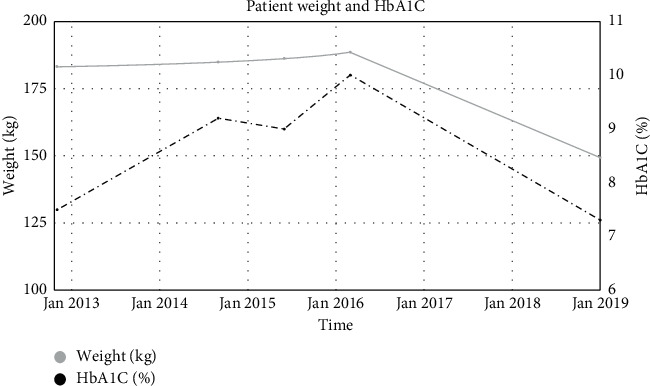
Patient weight and hemoglobin A1C graphed over time.

In March 2016, the patient was seen by endocrine surgery for consideration of a laparoscopic left adrenalectomy for Cushing's syndrome. A repeat CT showed the tumor had increased in size to 5.8 cm and was still partially composed of macroscopic fat and consistent with a myelolipoma (Figure 1). BMI was 65.1 kg/m^2^ (weight = 188.7 kg) and HbA1C was 10.0%. She underwent an uncomplicated laparoscopic adrenalectomy in October 2017. The case was performed via a standard transabdominal approach. The patient had a generous amount of intraabdominal fat which was mobilized with the colon and spleen from the left adrenal gland. She required an increased pneumoperitoneum pressure to facilitate exposure (17 cm of water). The remainder of her case and postoperative course were unremarkable. On surgical pathology, she was noted to have a 6.7 cm adrenal collision tumor comprised of a 4 cm adrenocortical adenoma and a 2.7 cm myelolipoma (Figure 2). Postoperatively, the patient was placed on corticosteroid replacement, which was tapered over the course of a year and a half without significant symptoms of adrenal insufficiency. 24 hour urine cortisol following discontinuation of steroids normalized to 7.6 *µ*g/24 hours. Her weight decreased to 149.4 kg with a BMI of 51.6 and HbA1c of 7.3% (Figure 3).

## 2. Discussion

We present the case of a 45-year-old woman with a radiographically diagnosed myelolipoma and ACTH independent Cushing's syndrome that underwent laparoscopic left adrenalectomy with surgical pathology showing a collision tumor with a 4 cm adrenocortical adenoma and 2.7 cm myelolipoma. Following surgery, her weight and diabetes improved and 24 hour urine cortisol normalized.

Incidentally discovered adrenal tumors, adrenal incidentalomas (AIs), are common, occurring in 3–7% of abdominal imaging performed for other reasons [1]. The majority of AIs are benign adrenocortical adenomas [7]. It is generally accepted that most AIs should undergo evaluation for the autonomous secretion of cortisol, catecholamines, and aldosterone (in hypertensive or hypokalemic patients) [1]. About 10–12% of AIs are cortisol secreting [7].

The presence of macroscopic fat on CT in an AI is considered virtually diagnostic of myelolipoma [8]. Adrenal myelolipomas are uncommon tumors that are composed of mature adipose and myeloid tissue and thought to be benign tumors which are not capable of autonomous hormone production [9]. It is generally accepted that myelolipomas do not warrant an evaluation for autonomous hormone production. The diagnosis of myelolipoma is made radiographically by the presence of an adrenal tumor containing macroscopic fat [10], but our group has previously reported that the accuracy of this diagnosis may depend on the amount of macroscopic fat seen in the tumor [11]. While adrenal myelolipomas are hormonally inactive, there is an increased association of myelolipomas and multiple endocrine disorders, including Cushing's syndrome. Although the exact etiology is unknown, one theory suggests a hormonally induced transformation of adrenal tissue into myeloid and lipomatous cells [12, 13].

Alternatively, others have suggested adrenal tissue necrosis may lead to myelolipomatous change [9, 10]. Both theories may account for the incidence of myelolipomas coupled with adrenal hyperplasia and Cushing's syndrome, as it has been reported [12, 14, 15].

Long-term hypercortisolism is associated with diabetes mellitus, hypertension, hyperlipidemia, and osteoporosis. Laparoscopic adrenalectomy has been shown to improve outcomes over nonsurgical therapy [16] and is the recommended treatment for patients with unilateral cortisol-secreting adrenal tumors [17]. Long-term autonomous secretion of cortisol from an adrenal tumor leads to suppression of the contralateral adrenal gland. Therefore, patients undergoing adrenalectomy for unilateral hypercortisolism require postoperative glucocorticoid replacement until the recovery of their remaining adrenal gland. There can be a delayed recovery taking up to 24 months [18].

Only five other case reports have chronicled a combination of an adrenal myelolipoma and functioning adrenal adenoma in a patient with Cushing's syndrome [2–6] (Table 1). An additional case of an adrenal myelolipoma and a functioning adrenal adenoma in a patient with Conn's syndrome have also been described [17]. As with our patient, Lu et al. previously reported that the majority of myelolipoma-adrenocortical adenoma collision tumors were found in female patients, involved the left adrenal gland, and if functional, demonstrated hormonal profiles compatible with Cushing's syndrome [17].

## 3. Conclusion

Adrenal lesions with macroscopic fat on imaging are typically labelled as myelolipomas and may not undergo a functional evaluation. This case report demonstrates that clinicians should consider a functional evaluation of select patients with radiographically diagnosed myelolipomas as they may prove to be functional collision tumors.

## Figures and Tables

**Table 1:** Case reports of adrenal myelolipoma and functioning adrenal adenoma.

Case authors	Age	Gender	ACTH	Cortisol	Side	Tumor size (cm)	Symptoms
Vyberg et al. [16]	31	F	↓	↑	L	2.5	Weight gain, hair loss, ecchymoses
Selye et al. [13]	58	F	↓	↑	R	7 × 4 × 4	Diabetes mellitus
Jenkins et al. [15]	67	F	↓	↑	L	3.5 × 3 × 2	Diabetes mellitus
Su et al. [9]	29	F	↓	—	R	3.3 × 2.2 × 1.7	Transient hypertension
Guresci et al. [14]	1.5	F	—	↑	L	7 × 6 × 4.5	Hypertension, weight gain, excessive body hair growth

## Data Availability

No data were used to support this study.
